# Pandemic punch: SARS-CoV-2 hits pancreas

**DOI:** 10.1038/s41392-024-01807-2

**Published:** 2024-04-16

**Authors:** Ninel Azoitei, Sandra Heller, Alexander Kleger

**Affiliations:** 1 Institute of Molecular Oncology and Stem Cell Biology, Ulm University, Ulm, Germany; 2 Division of Interdisciplinary Pancreatology, Clinic of Internal Medicine I, University Hospital Ulm, Ulm, Germany

**Keywords:** Infection, Cell biology

The COVID-19 pandemic has profoundly impacted global health systems, revealing the extensive repercussions of SARS-CoV-2 beyond respiratory symptoms. Indeed, traditionally considered a respiratory virus, SARS-CoV-2’s showcased the ability to target multiple organ systems including pancreas, an organ crucial for regulating blood sugar levels and aiding digestion.

**Fig. 1:**
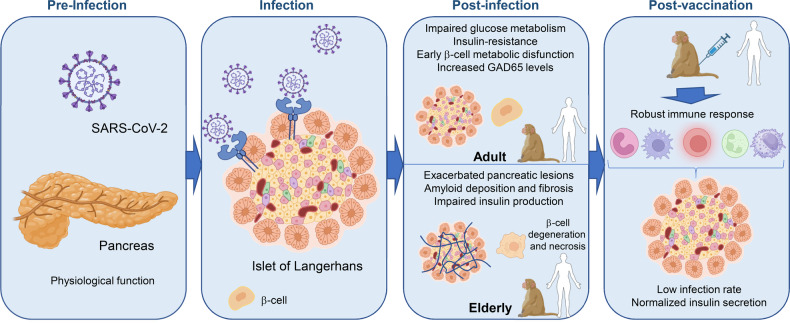
SARS-CoV2 hits pancreas. SARS-COV-2 virus infects pancreatic cells and disrupts their function in non-human primates. Following infection, elderly animals show worsened pancreatic damage, leading to increased risk of diabetes. Vaccination triggers a robust immune response, potentially protecting the pancreatic cells. The findings can be extrapolated to humans. The figure was created using the online tool BioRender (https://app.biorender.com/illustrations/)

Qin et al.‘s article titled ‘*Infection with SARS-CoV-2 can cause pancreatic impairment*’^1^ published in Signal Transduction and Targeted Therapy, sheds light into the mechanisms and implications of pancreatic involvement in COVID-19. This study represents a significant milestone in understanding the interplay between COVID-19 and metabolic disorders such as diabetes (Fig. 1).

The authors employed a retrospective analysis conducted on non-human primates (NHPs) infected or vaccinated and subsequently infected with SARS-CoV-2, and provide compelling evidence of the virus’s profound impact on pancreatic physiology. In adult monkeys, the study reveals that SARS-CoV-2 infection can significantly impair glucose metabolism and pancreatic function. The virus directly infects various types of pancreatic cells, including both exocrine and endocrine cells, leading to adverse effects on pancreatic physiology, consistent with previous reports.^2^ These effects are evidenced by inflammation, degeneration, and increased levels of GAD65, a major autoantigen associated with insulin-dependent diabetes. Despite these effects, severe damage to endocrine cells did not induce hyperglycaemia or diabetes in adult NHPs. Indeed, while a recent preprint shows that, similar to patients, not all NHPs developed hyperglycaemia or displayed elevated serum levels of insulin, the underlying reasons remain poorly understood with the only reasoning referring to variability in the viral entry protein expression.^2,3^ However, SARS-CoV-2 infection resulted in insulin resistance and glycometabolic dysfunction, evidenced by increased fasting C-peptide levels. This phenomenon is also observed in some COVID-19 survivors, suggesting a potential link between C-peptide levels and disease severity. Insulin resistance in NHP models seems minimally linked to pancreatic injury, but likely originates from impaired peripheral tissues such as adipose tissue and liver, driven by direct viral infection. These findings imply that COVID-19-related insulin resistance may primarily result from peripheral tissue dysfunction, rather than pancreatic or β-cell impairment. Conversely, in elderly monkeys, SARS-CoV-2 infection exacerbated pancreatic lesions and metabolic disorders, significantly increasing the risk of diabetes, suggesting an age-dependent vulnerability to COVID-19-induced pancreatic dysfunction but also delivering an explanation for the conflicting results reported by several studies employing human islets from various aged donors.^2,4^ Qin et al. propose several mechanisms through which SARS-CoV-2 infection may harm pancreatic β-cells in elderly monkeys, including direct cellular infection, increased vascular permeability, amyloid deposition, and activation of stellate cells leading to changes in the islet microenvironment. Particularly, amyloid deposition and fibrosis observed in the pancreatic tissue may exacerbate microscopic tissue changes associated with normal aging. These factors collectively contribute to β-cell degeneration and necrosis, potentially leading to reduced insulin secretion and increased risk of diabetes, both insulin-resistant and insulin-dependent types.

The increased risk of insulin-resistant diabetes is supported by data integrating proteomics and metabolomics analyses. Within others, the study shows that enriched insulin resistance pathways and aberrations in fasting C-peptide levels underscore the systemic metabolic upheaval induced by viral infection. The loss of β-cells, amyloidosis, and necrosis further underscore the deleterious impact on pancreatic tissue integrity, culminating in severe glycometabolic dysfunction.

Crucially, Qin et al. also illuminate rays of hope upon showcasing the protective effect of SARS-CoV-2 vaccination on pancreatic health. While some case studies linked mRNA COVID-19 vaccines to mild pancreatitis, a recent prospective cohort study did not measure increased risk even in the context of underlying pancreatic autoimmune disease. No significant changes were observed in vaccinated monkeys’ behaviour or pancreatic enzymes. At the same time, inflammation induced by vaccination may protect against pathogenic microorganisms, with B and T cells increasing in vaccine-stimulated germinal centres and which could ultimately migrate to the pancreas via capillaries and lymphatics to mount a local immune response against SARS-CoV-2, potentially reducing pancreatic infections.^5^ Despite transient elevation in some pancreatic markers, glucose and C-peptide levels did not significantly increase post-vaccination, suggesting no persistent pancreatic damage. Here, multi-omics analysis showed improvement in glycolipid metabolism and insulin resistance. Overall, SARS-CoV-2 infection-associated pancreatic impairment appears age-related, while vaccination potentially protects against pancreatic infection.

The implications of this study extend far beyond the realm of animal models. With mounting evidence pointing towards an enhanced risk of diabetes post-COVID-19, particularly among older individuals, it becomes imperative to extrapolate these findings to human populations. The insidious nature of pancreatic impairment demands heightened vigilance in diabetic management, necessitating tailored interventions to mitigate the substantial burden of metabolic disorders in the wake of the pandemic.

While we still navigate the uncharted waters of the post-COVID-19 era and face emergence of new SARS-CoV-2 strains, Qin and colleagues seminal research becomes paramount for future investigations having as a focal point the battle against metabolic derangements in general, and of the pancreas in particular. This study offers the scientific community not only valuable insights into the mechanisms and consequences of SARS-CoV-2 infection on pancreatic function but also paves several avenues for further research and indicates implications for clinical practice. Thus, in the efforts towards *clinical translatability*, the findings from animal models should be further validated in human studies to confirm the relevance and extent of pancreatic impairment in COVID-19 patients. Longitudinal studies tracking pancreatic function in COVID-19 survivors could provide valuable insights into the long-term effects of the virus on metabolic health. This study reveals an age-dependent vulnerability. Indeed, understanding of *age-dependent susceptibility* to pancreatic dysfunction post-COVID-19 is crucial. Further research should focus on elucidating the molecular mechanisms underlying this vulnerability, potentially leading to age-specific therapeutic interventions. We learned here that vaccination potentially protects against pancreatic infection which warrants continued investigation and improvement of *vaccine efficacy*. In this endeavour, longitudinal studies assessing pancreatic function in vaccinated individuals, particularly in high-risk groups such as the elderly, can provide insights into vaccine efficacy beyond preventing respiratory symptoms. This will be successful only upon implementation of novel *therapeutic strategies* relying on minute knowledge of the intimate molecular mechanisms unfolding during pancreatic impairment. Particularly, therapies aimed at preserving pancreatic β-cell function or improving peripheral tissue insulin sensitivity could reduce the risk of diabetes post-COVID-19. Lastly, this study underscores the importance of integrating pancreatic health assessment into post-COVID-19 care protocols, especially for individuals with pre-existing metabolic conditions. *Health care systems* across the globe need to improve addressing the increased burden of diabetes and metabolic disorders in the aftermath of the pandemic.

In conclusion, the study contrasts the effects of SARS-CoV-2 infection and COVID-19 vaccines on the pancreas. It suggests that SARS-CoV-2-related pancreatic impairment may be age-related, while vaccination could offer protection against pancreatic infection. These findings provide insights into COVID-19-related pancreatic disorders, highlighting the potential benefits of vaccination in preventing pancreatic complications.
